# Myeloid Neoplasias: What Molecular Analyses Are Telling Us

**DOI:** 10.5402/2012/321246

**Published:** 2012-09-27

**Authors:** Luciana M. Gutiyama, Diego F. Coutinho, Marina V. Lipkin, Ilana R. Zalcberg

**Affiliations:** Laboratório de Biologia Molecular, Centro de Transplantes de Medula Óssea, Instituto Nacional do Câncer (INCA), Praça da Cruz Vermelha 23, 6° Andar, 20230-130 Rio de Janeiro, RJ, Brazil

## Abstract

In the last decades, cytogenetic and molecular characterizations of hematological disorders at diagnosis and followup have been most valuable for guiding therapeutic decisions and prognosis. Genetic and epigenetic alterations detected by different procedures have been associated to different cancer types and are considered important indicators for disease classification, differential diagnosis, prognosis, response, and individualization of therapy. The search for new biomarkers has been revolutionized by high-throughput technologies. At this point, it seems that we have overcome technological barriers, but we are still far from sorting the biological puzzle. Evidence based on translational research is required for validating novel genetic and epigenetic markers for routine clinical practice. We herein discuss the importance of genetic abnormalities and their molecular pathways in acute myeloid leukemia, myelodysplastic syndromes, and myeloproliferative neoplasms. We also discuss how novel genomic abnormalities may interact and reassess concepts and classifications of myeloid neoplasias.

## 1. Introduction 

The discovery of informative cancer-related molecular biomarkers will provide more effective treatments and enhance the development of new targeted drugs. The recent advent of high-throughput (HT) technologies is speeding up and improving procedures for pursuing this goal. The number of new HT techniques is already very high, and the amount of available data is even more impressive. The central question now is how to analyze and validate all these data. The main problem relies on adopting a consensus strategy for the most suitable procedures based on the choice of candidate genes and markers in the heterogeneous panel of most hematological malignancies. 

A more comprehensive approach in carcinogenesis, based on sequencing of cancer exomes, has been put forward together with gene expression and copy number variation analyses. These approaches have been used for studying different cancer types, like glioblastoma, pancreatic and breast tumors [1–5], and shed light on the complexity of their genetic profiles due to the high number of somatic variations, even between tumors of the same cancer type. Interestingly, when the functions of mutated genes were analyzed, biological pathways turned out to be quite convergent. These findings indicated that accumulation of somatic mutations is responsible for driving cells to carcinogenesis. 

Next-generation sequencing (NGS) has reduced costs and time for entire genome analyses with deep coverage [6]. The whole-genome sequencing included for the HT analysis relevant conserved elements from noncoding regions. Knowledge of these new features, like noncoding RNAs (ncRNAs) and micro-RNAs (miRNAs), has added valuable data to HT studies, and reminiscent gaps are now being elucidated. NGS enables the identification of structural and copy number variation such as mutations, deletions, insertions, and duplications. Many complete cancer genomes have been resequenced for accurately discriminating polymorphic variants from potential malignant somatic mutations by comparing normal and tumor genome from the same patient. Technical barriers are surpassed; we are close to have personal medicine as a reality, and what we need now is to focus on the right target. 

In this postgenomic era, several cancer HT studies have been carried out, a reason why guidelines are needed for standardizing clinical data, disease stage, sequencing techniques, and analysis, as well as integrating massive sequence data. Due to the vast amount of data generated by NGS, join efforts are being employed for interpreting results in a context of clinical significance. Plenty of initiatives have been put forward with this purpose; in 2004, the Sanger Institute founded the Cancer Gene Census, a catalogue of genes listing mutations occurring at a higher frequency than normally expected [7]. The following year, the National Human Genome Research Institute and the National Cancer Institute launched The Cancer Genome Atlas (TCGA), a catalogue of genomic alterations involving more than 20 common types of cancer compiled by more than 100 researchers. All data generated by TCGA are available at the TCGA site (https://tcga-data.nci.nih.gov/tcga/tcgaHome2.jsp). The National Institutes of Health (NIH) also launched the Childhood Cancer Therapeutically Applicable Research to Generate Effective Treatments (TARGET) Initiative (http://target.cancer.gov/) for identifying therapeutic targets for childhood cancers, including acute lymphoblastic leukemia (ALL). In 2008, the International Cancer Genome Consortium (ICGC) was created to standardize genomic techniques and procedures for studying human cancers (http://www.icgc.org/). Another publication, the Catalogue of Somatic Mutations in Cancer (COSMIC), was launched to compile somatic mutations in tumors [8]. 

Besides revealing novel biomarkers, recent use of HT genome sequencing in acute myeloid leukemia led to a relevant finding of clonal evolution during relapse showing that, following chemotherapy, a foundational cell clone survivor of treatment or a new subclone acquired new mutations and expanded during relapse, demonstrating that therapy itself might affect clonal evolution and relapse. This study showed that chemotherapy induces DNA damage, leading to drug resistance in emergent surviving tumor cells or new subclones causing relapse. This finding highlights that eradication of the founding clone and all of its subclones will be required to achieve cures [9].

## 2. Acute Myeloid Leukemia (AML)

AML is characterized by a maturational arrest of bone marrow cells in early stages of myeloid development [10, 11]. AML is a relatively rare condition affecting mainly adults, although it was also detected in children [12]. The Brazilian National Cancer Institute estimates that AML affects 4 in 100.000 individuals, being more frequent in men than women [13]. 

Different factors have been associated to an increased risk of AML: previous hematological disorders, hereditary syndromes, and environmental and drug exposures, although most patients who present *de novo* AML have no identifiable risk factor. AML is a highly heterogeneous disease, since patients may show different biologic and clinical presentations among which genetic and epigenetic lesions are the most important [14, 15]. The World Health Organization (WHO) incorporated molecular markers to the latest AML classification, leading to the implementation of direct strategies for targeted treatments based on the mutational spectrum of this malignancy [16].

Cytogenetic analysis defines three major groups based on risk of therapy failure [17, 18]. Molecularly, the favorable risk group is characterized by alterations of the core binding factor (CBF) or the retinoic-acid receptor alpha gene (*RARA*) [19]. Patients at higher risk show loss of chromosome 5 or 7, as well complex karyotypes [17]. A clinical and biological heterogeneous group of intermediate risk comprises 50% of AML cases, mostly with a normal karyotype (CN-AML). CN-AML patients have been categorized following the incorporation of their mutational status respective to *FLT3 *(fms-related tyrosine kinase 3)*, NPM1* (nucleophosmin), and *CEBPA *(CCAAT/enhancer-binding protein (C/EBP), alpha) genes in the WHO 2008 classification [17–24]. Other frequent mutations recently described in AML affect the oncogenes *N*- and *K-Ras *and other genes like* MLL *(mixed-lineage leukemia)*, RUNX1* (runt-related transcription factor 1), *KIT *(feline sarcoma viral oncogene homolog), and *WT1* (Wilms' tumor 1). 

*FLT3, NPM1*, and *CEBPA* alterations have become important prognostic factors for stratifying CN-AML and can also affect the initial course of AML attributable to the presence of other markers [25]. *FLT3* mutations can affect two different regulatory domains: the juxtamembrane (JM) and the tyrosine kinase domain (TKD), leading to constitutive activation of FLT3. Internal tandem duplication (ITD) of the JM domain encoded by *FLT3 *exons 14 and 15 is found in 15–25% of AML patients and associated with a poor outcome. *ITD-FLT3* is more frequent in CN-AML and in patients carrying *t*(15; 17), *t*(6; 9), and *NPM1* mutation [26]. Large-scale studies demonstrated that *ITD-FLT3* has a negative impact, altering the favorable prognostic value of *PML-RARA* and *NPM-1 *mutation, when coexisting in AML [25].

TKD mutations are found in 6–10% of all AML patients [27]. *FLT3-TKD* is also more prevalent in patients with CN-AML and those carrying *NPM1* mutations or inv(16). Unlike *ITD, FLT3-TKD* has not been confirmed as a prognostic marker. Several studies have shown differences between these two kinds of mutations, differently affecting *FLT3* activation and the downstream signaling pathways, especially STAT5 (activator of transcription 5) [25, 26].

*CEBPA* mutations are found in *∼*10% of CN-AML, affecting DNA binding and dimerization with other CEBP family members [28]. They are associated with a favorable prognosis and comprise N- and C-terminal mutations. The majority of AML patients carry both kinds of *CEBPA* mutations, usually on different alleles [29]. Deregulation of CEBPA function can result from genomic mutations, transcriptional and posttranscriptional suppression, or inactivation by phosphorylation. Consequently, absence of* CEBPA* mutations does not always result in loss of protein function [30].

*NPM1 *mutations are the most frequent ones in CN-AML, in *∼*50% of patients. NPM1 binds to several proteins and is a known regulator of TP53 function in response to cellular stress factors. Mutation in *NPM1 *exon 12 results in loss of its nuclear localization signal. The altered protein concentrates in the cytoplasm, where it dimerizes to wild-type NPM1, blocking its activity in the nucleus [31]. Despite all efforts to find targeted therapies for recurrent abnormalities in AML cells, leukemic subclones seem to acquire resistance due to the presence of additional or secondary molecular lesions. One case was recently reported under treatment with the FLT3-specific inhibitor quizartinib/AC220 [32]. As previously discussed, *FLT3-TKD* mutations have not shown to be of prognostic value for standard chemotherapy. However, unlike *ITD-FLT3* mutations, they confer resistance to quizartinib and may appear as secondary mutations during therapy. *CEBPA* mutations, associated with a better outcome at diagnosis, may, however, confer resistance against TK-induced differentiation. Taken together, these data indicate that the prognostic impact of genetic abnormalities may vary according to the therapeutic approach [30]. 

Although AML stratification may discriminate between different outcomes, many patients still lack significant markers of prognostic significance. Besides, different response to therapy occurs among patients of the same risk groups. With the goal of refining prognosis for AML, the search for additional alterations was carried out with integrated genetic profiling techniques [25]. In the last three years, with whole-genome sequencing approaches, several novel mutations have been identified in genes involved in epigenetic (*IDH1* and 2-isocitrate dehydrogenases; *TET2-*tet methylcytosine dioxygenase 2; *DNMT3*—DNA (cytosine-5-)-methyltransferase 3 alpha) and posttranscription regulation (miR-155, miR-29, and miR-146), pointing to the complex nature of AML [25]. 

*DNMT3A* mutations appear to be a common alteration in adult AML patients, with an overall prevalence of 20%. DNMT3A is involved predominantly in *de novo* methylation. Mutations affect conserved functional regions of this protein, the majority of them located in the catalytic domain. Moreover, *DNMT3A*—as well as *MLL *mutations—defines a biologic subgroup of AML patients typically presenting a myelomonocytic or blastic morphology and marked leukocytosis. This molecularly characterized group might benefit from intensive induction chemotherapy with high doses of daunorubicin [33].

miRNA genome-wide analyses revealed signatures associated to specific subgroups with homogeneous cytogenetic and clinical outcome. The role of miRNA in myeloid leukemogenesis is still not completely understood, but the association between miRNA and mRNA has provided interesting insights. Different independent miRNA-expression profiles indicated that AML patients with *t*(8; 21), inv(16), and *t*(15; 17) showed unique miRNA signatures capable of discriminating this group from other AML subtypes. miR-126/126* was specifically overexpressed in both *t*(8; 21) and inv(16) AML, while miR-224, miR-368, and miR-382 were almost exclusively upregulated in *t*(15; 17) AML. Upregulation of miR-155 in patients with *ITD-FLT3* has also been reported, suggesting an association between miR-155 and increased proliferation [34, 35].

**Figure 1:**
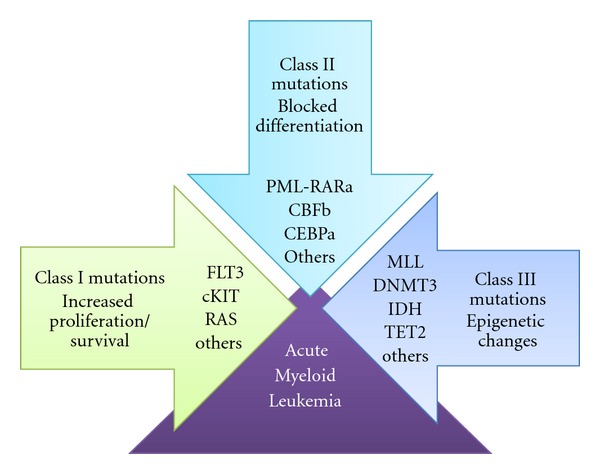
Molecular events related to AML.

In summary, about ten cytogenetic or molecular abnormalities are consistently present in every AML genome, reinforcing the postulation that AML is a multistep malignancy. The recent reassessment of the two-hit-model theory [36] indicates at least three types of events associated to malignant transformation: class I mutations—affecting proliferation and survival, class II mutations—blocking normal differentiation, and class III alterations—interfering with epigenetic regulation (Figure 1).

## 3. Myeloproliferative Neoplasms and Myelodysplastic Syndrome

Myelodysplastic syndromes (MDSs) and myeloproliferative neoplasms (MPNs) are hematological disorders characterized by marrow hypercellularity with an abnormal blood cell count, both capable of transforming to AML [37, 38]. MDS affects mostly adults over 70 of age although the incidence of childhood and juvenile cases has shown to be increasing [39].

The WHO classification considers as classical, *BCR-ABL*-negative MPN disorders three different diseases with clinical and biological similarities: polycytemia vera (PV), essential thrombocythemia (ET) and primary myelofibrosis (PMF). A major breakthrough in the understanding of molecular MPN pathogenesis has been achieved with the identification of the V617F mutation in the Janus kinase 2 gene (*JAK2*V617F). 

*JAK2*V617F-activating mutation is the most prevalent abnormality observed in *BCR-ABL*-negative MPN, in virtually all cases of PV and in about 50% of ET and PMF. The JAK2V617F mutation represents the most important factor for understanding molecular mechanisms underlining MPN pathogenesis, contributing to diagnosis and management of patients.

The etiology of MDS and MPN is still unknown, but it likely involves DNA damage of hematopoietic stem cells [40]. Different myeloid malignancies, including AML, share the same alterations in genes like *IDH1, IDH2, ASXL1 *(additional sex combs like 1),* EZH2 *(enhancer of zeste homolog 2 gene),* RUNX1, DNMT3A, TET2, p53*, and *CBL *(Casitas B-cell lymphoma gene), although unlike the JAK2V617F mutation, none of these markers can alone define diagnosis for any specific myeloid entity. The frequency of mutations in these genes varies among MPN, MDS, and AML [25, 41, 42] and also with age. Compared with adult AML, the incidence of *TET2* mutations in pediatric cases seems to be lower (8–19% versus 3.8%) [43, 44]. 

Somatic mutations identified to date do not seem to be acquired in any preferred order, and disease-initiating events remain to be identified [45]. The fact that one genetic event (JAKV617F) is associated to at least 3 different phenotypes and that *IDH1, IDH2, ASXL1, RUNX1, DNMT3A, TET2, p53* and *CBL *mutations might be present in AML, MDS or MPN suggests that different molecular interactions between gene products result in specific malignant profiles. These could lead to different patterns, with a predominantly increased proliferation (MPN), ineffective hematopoiesis (MDS), or with similar levels of altered proliferation and differentiation (AML).

**Figure 2:**
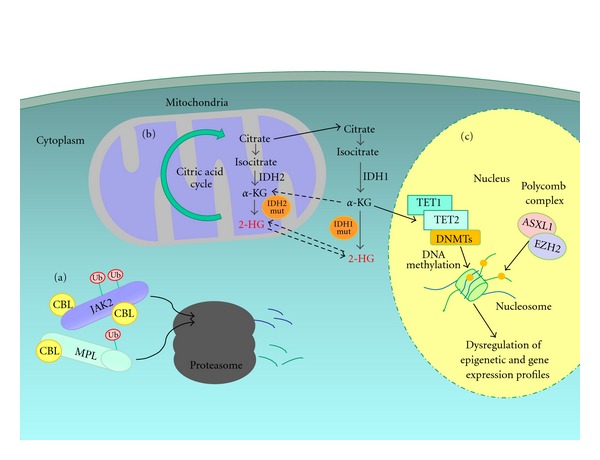
Molecular-metabolic pathways involved in myeloid neoplasias. (a) MPL and JAK2 cytoplasmic levels are controlled by ubiquitin (ub) CBL activity inducing protein degradation via proteasome. (b) IDH1/2 wild-type enzymes convert isocitrate to *α*-ketoglutarate (*α*-KG), a TET2 cofactor. IDH1/2 mutations catalyze *α*-KG conversion to 2-hydroxyglutarate (2-HG) oncometabolite. (c) Alterations in TET2 and DNMT enzymes lead to methylation deregulation leading to hypomethylation. ASXL1 and EZH2 loss-of-function affect chromatin structure favoring a relaxed state. These modifications increase gene transcription.

MDS and MPN genes belong to two major pathways, intracellular metabolism and epigenetic regulation (Figure 2). Recently, other genes like *SRSF2 *(serine/arginine-rich splicing factor 2),* ZRSR2* (zinc finger-CCCH type), and *U2AF1 *(U2 small nuclear RNA auxiliary factor 1) have been found to be involved in the splicing pathway in MDS [46]. 

The intracellular metabolism pathway includes enzymatic families acting in protein degradation and the citric acid cycle like CBL, IDH1, and IDH2. CBL proteins are biomolecules with ubiquitin ligase activity, an important process of protein degradation in proteasomes. Tyrosine kinases and cytokine receptors, including, JAK2 and MPL proteins, respectively, are CBL targets. CBL mutations alter protein degradation and, consequently, intracellular signaling. In the citric acid cycle, IDH1 and IDH2, encoded by *IDH1* and *IDH2 *genes, catalyze the conversion of isocitrate to *α*-ketoglutarate, a TET2 regulator (enzyme cofactor), resulting in a decreasing of 5-hydroxymethylcytosine-(5hmC-) methylation pathway. Mutations in exon 4 of *IDH1 *and* IDH2* result in neomorphic enzymes, responsible for production of the oncometabolite (R)-2-hydroxyglutarate from *α*-ketoglutarate. Thus, *IDH1 or IDH2* mutations in myeloid malignances may induce DNA damage or epigenetic alterations. These latter may occur by mutations leading to loss of function of TET2, impairing conversion of methylcytosine (5mC) to 5-hydroxymethylcytosine (5-hmC) that in turn results in a lower level of methylation [47]. The lower status of promoter methylation leads to increased transcription.

The polycomb complex is a multiprotein PRC1-like complex, a complex class required to maintain the transcriptionally repressive state of many genes, remodeling and modifying histones by methylation or acetylation. Two enzymes involved in the polycomb complex are coded by *EZH2* and *ASXL1,* and mutations affecting these genes have been reported in several myeloid malignances [55–58]. Transcription factors involved in myeloid differentiation can also be quantitatively modulated by epigenetic events. This is the case of *RUNX1*, often mutated in MDS, MPN, and AML [59]. This gene acts as a transcription factor with a major role in myelopoiesis, regulating the expression of GM-CSF, G-CSF, CD11a, MPO, mast cell protease 6, and neutrophil elastase. *RUNX1* mutations are associated with blocked myeloid differentiation, a likely explanation for the presence of blasts in *de novo* AML and AML/SMD or MPN [53]. 

### 3.1. Splicing and MDS

Recently, whole-exome sequencing analysis of MDS patients showed genetic alterations in the splicing machinery. Mutations in spliceosomal genes, leading to aberrantly spliced mRNA, are apparently restricted to MDS [50]. Different mRNA isoforms of variable size would be expected to be formed, with longer transcripts affecting chromatin structure by favoring a more relaxed status and higher transcriptional levels.

Somatic mutations at the RNA splicing factor 3b subunit 1 (*SF3B1*) coding gene are recurrent in patients with MDS with ring sideroblasts (MDS-RSs), like in refractory anemia (RARS) and refractory cytopenia with multilineage dysplasia (RCMD-RS) [46]. Mutations at *SF3B1* are found in *∼*70% of MDS-RS patients, representing a putative biomarker of the disease. *SRSF2*,* ZRSR2*, and *U2AF1 *mutations show frequencies of *∼*8–30% in chronic myelomonocytic leukaemia (CMML) and MDS-RS patients [46]. The discovery of mutations affecting the splicing machinery represented a significant breakthrough for understanding the molecular complexity of MDS. Despite these findings, these mutations are not always present in all MDS patients, and validations are still under way before they can be used as reliable biomarkers.

## 4. Conclusions

The contribution of genetics and genomics to the diagnosis of myeloid disorders has been extremely valuable (Table 1). HT techniques are becoming more accessible, less costly, and consolidated, while analytic methods and algorithms are providing more accurate results. We have now overcome several technical barriers for a successful translational research. Present challenges should be canalized for adopting strict criteria in selecting adequate samples of well-characterized clinical entities, disease stages, and therapeutic response. A holistic biological approach is necessary for processing vast amounts of data for understanding the profoundalterations involved in myeloid neoplasias described at the genetic and epigenetic levels. The understanding of molecular metabolic pathways provided specific profiles presently used for diagnosis and stratification, establishing clonality and distinguishing MPN, MDS, and MDS/MPN, from reactive conditions. 

However, despite their relevance for effectively targeting therapies, we are still looking forward to a more comprehensive application of these findings in the treatment of myeloid leukemias. 

## Figures and Tables

**Table 1:** Brief summary of novel genetic and epigenetic markers for AML, MDS, and MPN.

Study [ref#]	No. of patients	Markers	Disorder
Milosevic et al. 2012 [48]	203	TP53, RUNX1, CBL, IDH1/2, NRAS, NPM1, and FLT3	MDS, AML, and MPN
Shih et al. 2012 [49]	—	TET2, IDH1/2, ASXL1, EZH2, and DNMT3A	MDS, AML, and MPN
Patel et al. 2012 [25]	398	TET2, ASXL1, DNMT3A, CEBPA, PHF6, WT1, TP53, EZH2, RUNX1, PTEN FLT3, NPM1, HRAS, KRAS, NRAS, KIT, IDH1, and IDH2	AML
Brecqueville et al. 2012 [41]	276	ASXL1, CBL, DNMT3A, IDH1/2, JAK2, MPL, NF1, SF3B1, SUZ12, and TET2	MPN
Yoshida et al. 2011 [50]	29	U2AF35, ZRSR2, SRSF2, and SF3B1	MDS
Zhang et al. 2012 [51]	53	SRSF2	MPN and AML
Langemeijer et al. 2011 [44]	151	TET2	AML
Schnittger et al. 2012 [52]	636	CBL, JAK2, and TET2	MPN
Thiede 2012 [53]	—	FLT3, NPM1, TET2, IDH1/2, and DNMT3A	AML
Bejar et al. 2011 [42]	439	ETV6, GNAS, RUNX1, TP53, EZH2, and NRAS	MDS
Cimmino et al. 2011 [54]	—	TET family	MDS, AML, and MPN
